# Advanced Non‐Thermal Processing: Combined Cold Plasma and Pulsed Electric Field for *E. coli* Inactivation in Milk

**DOI:** 10.1002/fsn3.71408

**Published:** 2025-12-29

**Authors:** Kimia Taki, Bahram Hosseinzadeh Samani, Shirin Ghatrehsamani

**Affiliations:** ^1^ Department of Mechanical Engineering of Biosystems Shahrekord University Shahr‐e Kord Iran; ^2^ Department of Agricultural and Biological Engineering The Pennsylvania State University University Park Pennsylvania USA

**Keywords:** cold plasma, *E. coli* inactivation, milk pasteurization, non‐thermal pasteurization, pulsed electric field

## Abstract

Milk, rich in proteins, vitamins, and bioactive compounds, is highly susceptible to microbial contamination by pathogens like *Escherichia coli* , posing risks to food safety. Conventional thermal pasteurization often degrades nutritional and sensory qualities. This study developed a non‐thermal ACP‐PEF system to inactivate *E. coli* while preserving milk's properties. Using Response Surface Methodology (RSM) with a Box–Behnken design, four parameters, electric field strength (5–10 kV/cm), exposure time (5–35 s), argon‐to‐air ratio (0–1), and nozzle angle (0°–90°) were optimized via Design‐Expert software. Optimal conditions (10 kV/cm, 35 s, ratio 0, angle 53.87°) achieved a 4.656‐log reduction (99.9978%) in *E. coli* . Quality analysis revealed minimal changes: pH (0.44%), fat (10%), protein (7%), lactose (7%), SNF (4%), and FFP (2%) reduction, compared to untreated milk, outperforming thermal pasteurization (reductions of 2%–20%). Color difference (ΔE = 0.81 ± 0.10) was insignificant. The ACP‐PEF system, combining electroporation and RONS, offers a scalable, energy‐efficient alternative for safe, high‐quality milk production.

## Graphical abstract

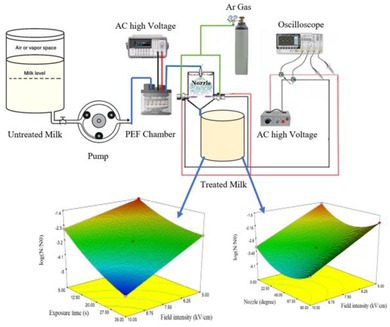

## 1 Introduction

Milk is a common part of people's diets all around the world because milk has protein, vitamins, minerals, and bioactive substances such as lactoferrin and immunoglobulins (Lambrini et al. 2021). Because it is wet and full of nutrients, germs like *Escherichia coli* , *Listeria monocytogenes* , and *Salmonella* spp. can easily get in, which poses risks to food safety and public health, especially for babies, the elderly, and people with weak immune systems (Luksiene 2021). Low‐temperature long‐time (LTLT) at 62°C for 15 min, high‐temperature short‐time (HTST) at 72°C–75°C for 15–30 s, and ultra‐high‐temperature (UHT) processing at 125°C–150°C for 2–3 s are conventional microbiological safety procedures (De Jong 2008). Milk loses its nutritional value and flavor as proteins break down, lipids oxidize, and taste and smell change (Hosseini et al. 2020) (Wang et al. 2024).

As thermal processing has its limits, non‐thermal procedures that keep milk's nutritional and sensory properties and stop microbial growth are becoming increasingly popular. PEF, ACP, HHP, and hydrodynamic cavitation can kill germs without hurting the quality of the product (Taki et al. 2025) (Dey et al. 2016). PEF releases high‐voltage electric pulses into the membranes of bacterial cells to stop them from working without heating them (Ghoshal 2023). At low temperatures and normal pressure, ACP makes reactive oxygen and nitrogen species (RONS) that break down bacterial structures in heat‐sensitive foods like milk (Misra et al. 2016). Venturi tube reactors kill microorganisms by popping bubbles and sending shock waves across the water (Asaithambi et al. 2019).

Researchers have looked at these technologies on their own, but when they are put together as a hurdle technology, they are able to kill germs at lower treatment intensities than PEF alone (Ding et al. 2021) (Gomez‐Gomez et al. 2021). Liquid‐phase plasma with hydrodynamic cavitation can eliminate germs in milk (Taki et al. 2025). There hasn't been much research on using ACP and PEF in a spraying system to pasteurize milk, notably its complicated mix of proteins, fats, and lactose.

This study built on and improved an atmospheric cold plasma‐pulsed electric field (ACP‐PEF) spraying system for pasteurizing milk, drawing on insights from Venturi tube reactors and liquid‐phase plasma (Taki et al. 2025). Milk is hard to process without heat since it has a high protein and fat content. To keep the quality and safety of the milk, the parameters need to be optimized. This study fills in the gaps by looking at how the system changes the total protein, lactoferrin, lactose, vitamin content, sensory qualities, and *E. coli* , which is a sign of microbial contamination. People want dairy products that are minimally processed, taste nice, are safe, and are good for them. This is why this work is necessary.

The study employs ACP and PEF to produce a dairy industry‐friendly alternative to thermal pasteurization that can be scaled up, saves energy, and maintains quality. This research creates a way to spray milk with atmospheric cold plasma (ACP) and pulsed electric field (PEF) to find the best PEF intensity, exposure time, argon‐to‐air ratio, and plasma jet‐nozzle angle to kill the most *E. coli* while keeping the milk's nutritional value compared to thermal pasteurization.

## 2 Materials and Methods

### 2.1 Design Combined ACP‐PEF System

**FIGURE 1:**
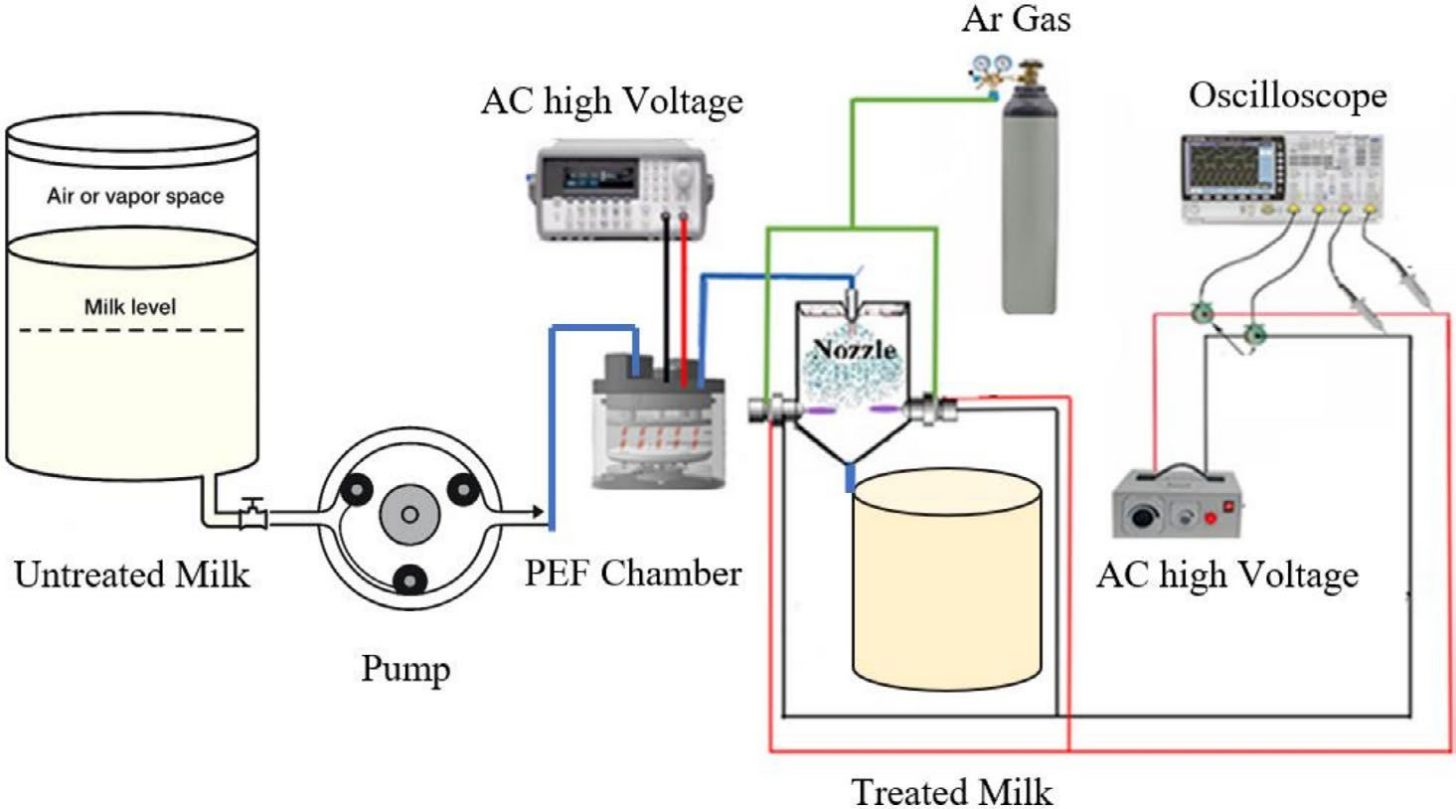
Schematic of combined ACP‐PEF system.

A custom‐designed combined ACP‐PEF system was developed for milk pasteurization. The system comprised two sequential treatment stages: a PEF unit followed by an ACP spraying chamber, leveraging hurdle technology to enhance microbial inactivation (Figure 1).

#### 2.1.1 Pulsed Electric Field Unit

The PEF system utilized in this research comprises four essential elements, as described by Jin and Zhang (2020): a pulse generation unit, a treatment chamber, a fluid management system, and monitoring and control devices. The pulse generation unit, which produces high‐voltage electric pulses, consists of a power source (24 kV), a capacitor array, and electronic switches (50 Hz). According to Mohamed and Eissa (2012), both alternating current (AC) and direct current (DC) power sources are suitable for charging the capacitor array. In this study, an AC power source was selected to optimize cost‐efficiency during system setup, maintaining high performance while minimizing initial investment. The sequence of applying PEF first, followed by ACP, was chosen based on the synergistic effects of electroporation from PEF enhancing the subsequent inactivation by RONS generated in ACP. This sequence has been reported to be more effective in pretest, as it maximizes the microbial inactivation while minimizing quality degradation.

#### 2.1.2 Atmospheric Cold Plasma Unit

The ACP system features a plasma generation setup with two coaxial electrodes: a central tungsten electrode (2 mm diameter, 8.5 cm length) and a copper ring electrode (1 mm thick, 1 cm long, 6 mm internal radius), separated by a 1 mm thick ceramic tube (5 mm inner radius, 9 cm length) acting as a dielectric layer. It utilizes an AC power source with a variable voltage range of 0–20 kV and a frequency range of 6–20 kHz, consuming approximately 50 W of power. The system employs a mixture of argon and air (adjustable argon‐to‐air ratio of 0–1) to generate reactive oxygen and nitrogen species (RONS) for bacterial inactivation, with two plasma jets integrated into a spraying system where the jet‐nozzle angle is adjustable from 0° to 90°. This configuration ensures effective microbial deactivation while maintaining the non‐thermal nature of the treatment, preserving the quality of sour cherry juice.

### 2.2 Microbial Inactivation Assessment

Sterilization was conducted on all samples of milk before inoculation to ensure the accuracy of the microbiological tests. The samples were autoclaved at 121°C and 15 psi for 15 min to eradicate any microbiological contamination that may have existed prior to sterilization. Subsequent to the sterilization procedure, the samples were deliberately inoculated with *E. coli* to evaluate the efficacy of the ACP‐PEF system in diminishing bacterial populations. To prepare the inoculum, *E. coli* colonies cultivated for 24 h were transferred into a sterile saline solution containing 0.9% sodium chloride.

A specified amount of the bacterial culture was combined with autoclaved milk at a 1:9 ratio (volume to volume) to provide a uniform initial microbial load. To assess the efficacy of the ACP‐PEF therapy, bacterial counts were evaluated both before and throughout the treatment. The viable plate count technique achieved the quantification of microorganisms. Five consecutive dilutions were conducted for each sample, followed by the injection of 1 mL from each dilution onto MacConkey Agar. The plates were maintained in an incubator at 37°C for 24 h to promote the proliferation of *E. coli* . After the incubation time, the colonies on each plate were enumerated to ascertain the bacterial quantity contained in the samples. To ensure the consistency and precision of the findings, each test was conducted three times (Hosseini et al. 2020).

### 2.3 Quality Parameter Analysis

Milk, a nutritionally rich and complex fluid, contains essential components such as fats, proteins, lactose, minerals, and various micronutrients, making it a vital dietary staple. Evaluating its quality attributes is critical for ensuring its nutritional value and safety. This study investigates the influence of a combined processing system on key milk quality parameters, including fat content, protein levels, lactose concentration, solids‐not‐fat (SnF), free‐fat portion (FFp), and pH.

#### 2.3.1 pH Measurement Methodology

The pH of milk samples was determined using a high‐precision digital pH meter (Consort C933 Electrochemical Analyzer), featuring a measurement range of 0–14 pH, a resolution of 0.01 pH, and an accuracy of ±0.01 pH. Calibration was performed using a three‐point method with standard buffer solutions (pH 4.00, 7.00, and 10.00) to ensure measurement reliability. The milk samples were equilibrated to ambient temperature, and the pH meter's electrode was thoroughly rinsed with distilled water before immersion in the sample. The pH value was then recorded directly from the meter's display.

#### 2.3.2 Analysis of Milk Composition

To evaluate the levels of fat, protein, and lactose, a MilkoScan Minor (LactoStar, Funke Gerber, Germany) was employed. This device offers an accuracy of ±0.02% and enables simultaneous measurement of multiple milk quality parameters. The instrument was powered on, and a milk sample was transferred into a designated container. The device's probe was then inserted into the sample, and the results for fat, protein, lactose, and other parameters were displayed on the screen for analysis.

#### 2.3.3 Color Analysis

Milk quality was assessed by measuring pH (Model pH −300, Mettler Toledo, Switzerland), viscosity (Model VT‐500, Brookfield, USA), and fat content (Gerber method). Total phenolic content (TPC) was determined using the Folin–Ciocalteu assay, with absorbance at 760 nm (Model UV‐2000, Shimadzu, Japan). Color was evaluated using a colorimeter (Model CR‐400, Konica Minolta, Japan), with total color difference (ΔE) calculated as in Equation (1).

(1) $$\Delta E=\sqrt{{\left(L_{0}-L^{*}\right)}^{2}+{\left(a_{0}-a^{*}\right)}^{2}+{\left(b_{0}-b^{*}\right)}^{2}}$$

where $L_{0},a_{0},b_{0}$ are untreated milk color parameters, and $L^{*},a^{*},b^{*}$ are treated milk parameters.

### 2.4 Experimental Conditions and Analysis

The ACP‐PEF system was tested at electric field intensities of $5\mathrm{kV}{\mathrm{cm}}^{-1},7.5{\text{kVcm}}^{-1}$, and $10\mathrm{kV}{\mathrm{cm}}^{-1}$, exposure times of 10s,20s, and 30 s, argon‐to‐air ratios of 0, 0.5, and 1.0, and jet‐nozzle angles of $0^{\circ },45^{\circ }$, and $90^{\circ }$. Control samples included untreated milk and thermally pasteurized milk ($72^{\circ }C$ for 15 s). Each condition was tested in triplicate.

Response Surface Methodology (RSM) with a Box–Behnken design was implemented using Design Expert software to evaluate the effects of electric field intensity, exposure time, argon‐to‐air ratio, and jet‐nozzle angle on microbial inactivation and quality parameters. A quadratic model was fitted (Equation 2).

(2) $$Y=\beta_{0}+\sum \beta_{i}X_{i}+\sum \beta_{\mathrm{ii}}X_{i}^{2}+\sum \beta_{\mathrm{ij}}X_{i}X_{j}+\varepsilon$$

where Y is the response, $\beta_{0},\beta_{i},\beta_{\mathrm{ii}},\beta_{\mathrm{ij}}$ are coefficients, $X_{i},X_{j}$ are independent variables, and ε is the error. ANOVA was used to assess significance $\left(p<0.05\right)$, with results reported as mean ± standard deviation.

## 3 Results and Discussion

In this study, a non‐thermal pasteurization system combining ACP and PEF was developed and optimized to inactivate *E. coli* in milk. Four independent variables, electric field strength (5–10 kV/cm), exposure time (10–30 s), argon‐to‐air gas ratio (0.3–1), and nozzle angle (0°–45°), were selected for process optimization. The effects of these variables on the reduction of *E. coli* in milk samples were investigated. Response Surface Methodology (RSM) and Design‐Expert software were employed to model and analyze the influence of these parameters on the pasteurization process.

### 3.1 Analysis Method and Test Optimization

To optimize microbial inactivation experiments and evaluate the effects of independent variables on *E. coli* inactivation in milk, a stepwise regression analysis was conducted using RSM with a Box–Behnken design in Design‐Expert software. This approach enabled precise modeling of the effects of independent variables on the non‐thermal pasteurization process. The results of the analysis of variance (ANOVA) for this process are presented in Table 1, demonstrating the significant influence of the variables and their interactions on microbial load reduction. According to Table 1, *p*‐values less than 0.1 indicate the statistical significance of the model factors at a 10% probability level. Consequently, the independent variables field intensity (A), exposure time (B), argon‐to‐air gas ratio (C), and nozzle angle (D) as well as the interaction effects of field intensity and exposure time (A × B), field intensity and nozzle angle (A × D), exposure time and nozzle angle (B × D), and argon‐to‐air gas ratio and nozzle angle (C × D), along with the quadratic effects of exposure time (B^2^), argon‐to‐air gas ratio (C^2^), and nozzle angle (D^2^), were found to be significant in the inactivation of *E. coli* in milk.

**TABLE 1:** Analysis of variance for the independent variable coefficient model.

Source	Sum of squares	Df	Mean square	*F*	*p*
Model	22.61172	11	2.055611	4550.546	< 0.0001
A‐field intensity	8.534533	1	8.534533	18893.06	< 0.0001
B‐exposure time	10.62201	1	10.62201	23514.15	< 0.0001
C‐Ar to air	0.064533	1	0.064533	142.8587	< 0.0001
D‐Nozzle Angle	0.371008	1	0.371008	821.3084	< 0.0001
AB	0.3844	1	0.3844	850.9538	< 0.0001
AD	0.004225	1	0.004225	9.352965	0.0080
BD	0.030625	1	0.030625	67.79516	< 0.0001
CD	0.003025	1	0.003025	6.696502	0.0206
B^2^	0.525597	1	0.525597	1163.525	< 0.0001
C^2^	0.49498	1	0.49498	1095.746	< 0.0001
D^2^	2.407722	1	2.407722	5330.022	< 0.0001
Residual	0.006776	15	0.000452		
Lack of Fit	0.006309	13	0.000485	2.079976	0.3711
Pure Error	0.000467	2	0.000233		
Cor Total	22.6185	26			

**FIGURE 2:**
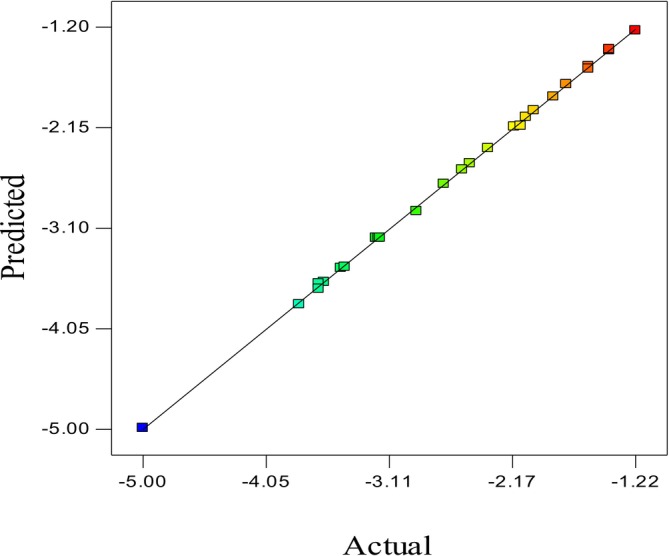
Experimental data versus modeling data.

Figure 2 presents the actual versus predicted data, a key tool for evaluating the accuracy of the statistical model in RSM. This figure illustrates the experimental values of *E. coli* microbial load reduction against the values predicted by the model. The distribution of data points along the *y* = *x* line indicates a high degree of agreement between the model and experimental data. The model exhibited a coefficient of determination (*R* ^2^) of 0.9997, a standard deviation of 0.021, and a coefficient of variation (CV%) of 0.80%, confirming its high accuracy and reliability.

Based on the analysis, two equations, actual and coded, were derived for the dependent variable of this study, namely the reduction in *E. coli* microbial load in milk. Equation (3), representing the actual equation, describes the relationship between the independent variables (field intensity, exposure time, argon‐to‐air gas ratio, and nozzle angle) and *E. coli* reduction using their real values. The coded equation, utilizing standardized values (scaled to a unitless range), was employed to analyze the relative effects and interactions of the variables (Equation 4).

(3) $$\begin{array}{cc} \log\left(N/\text{N0}\right)= & -3.19-0.84A-0.94B-0.07\text{3C}-0.18D\\ & -0.31\mathrm{AB}-0.03\text{2AD}-0.08\text{8BD}-0.02\text{8CD}\\ & +.30B^{2}+0.29C^{2}+0.63D^{2} \end{array}$$

(4) $$\begin{array}{cc} \log\left(N/\text{N0}\right)= & 0.80844-0.1590\text{0A}-0.047506B-1.2405\text{6C}\\ & -0.026691D-0.00826667\mathrm{AB}-0.00028888\text{9AD}\\ & -0.00012963\text{0BD}-0.00122222\mathrm{CD}+0.00131543B^{2}\\ & +1.1488{\text{9C}}^{2}+0.00031282{\text{6D}}^{2} \end{array}$$

Based on the coded Equation (4) and the coefficients of the independent variables, the absolute value of each coefficient indicates the extent of its influence on the reduction in *E. coli* microbial load in milk. According to this equation, the variables argon‐to‐air gas ratio (C), field intensity (A), exposure time (B), and nozzle angle (D) exhibited the greatest impact on *E. coli* inactivation, in that order. The negative coefficients of these variables signify their reductive effect on the microbial load, indicating that an increase in each variable leads to a decrease in the *E. coli* population.

### 3.2 Effect of ACP‐PEF Treatment on *E. coli* Inactivation in Milk

#### 3.2.1 Effect of Field Intensity and Exposure Time on Inactivation of *E. coli*

**FIGURE 3:**
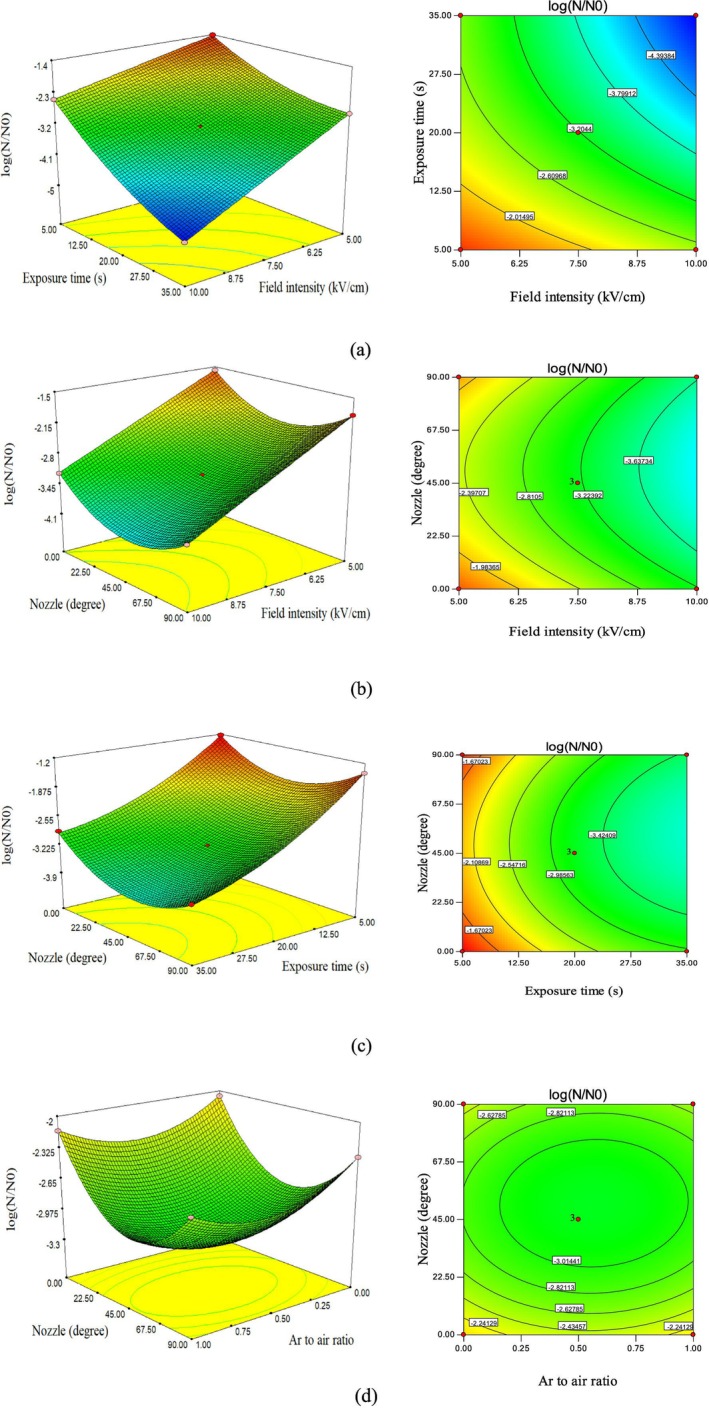
Effect of independent variable on *E. coli* reduction: (a) Exposure time‐Field intensity (b) Nozzle angle‐Field intensity (c) Nozzle angle‐Exposure time (d) Nozzle angle‐Ar air ratio.

Figure 3a illustrates the trends and effects of electric field intensity and plasma exposure time on *Escherichia coli* inactivation in milk using the combined cold PEF system. The variables were evaluated at three levels: electric field intensity (5, 7.5, and 10 kV/cm) and exposure time (5, 20, and 35 s). Based on the coded equation (Equation 4), the coefficient of electric field intensity (A) exhibited a greater influence on microbial load reduction compared to plasma exposure time (B). Analysis of Figure 3a revealed that increasing the electric field intensity from 5 to 7.5 kV/cm resulted in a logarithmic reduction from −2.24 to −3.21. Further increasing to 10 kV/cm led to a −4.03 logarithmic reduction.

The increase in electric field intensity in the PEF process plays an important role in microbial inactivation. A strong electric field intensity induces a high electrical potential across bacterial cell membranes, triggering electroporation. This phenomenon creates temporary or permanent pores in the cell membrane, allowing ions and molecules to penetrate, disrupting cellular functions, and ultimately leading to cell death. Previous studies, such as Ghoshal (2023), have confirmed that electric field intensity above 5 kV/cm significantly enhances electroporation rates in *E. coli* , as the applied energy is directly proportional to the field strength (Ghoshal 2023). In the combined ACP‐PEF system, this effect is amplified by the production of reactive oxygen and nitrogen species (RONS), such as ozone (O3) and hydroxyl radicals (·OH), which oxidize the PEF‐damaged cell membranes, accelerating inactivation. For instance, at 10 kV/cm, a logarithmic reduction exceeding 4 units highlights the strong synergy between the two technologies, achieved without elevating temperature or compromising milk's sensory qualities.

These findings align with Sitzmann et al. (2016), who demonstrated that combining PEF with other non‐thermal methods, such as plasma, can significantly enhance inactivation efficiency (Sitzmann et al. 2016). This plot underscores that electric field strength is the primary factor in improving process efficacy, and its optimization can lead to effective non‐thermal pasteurization while preserving milk's sensory attributes.

Also, analysis of Figure 3a revealed that increasing the exposure time from 5 to 20 s resulted in a logarithmic reduction from −1.95 to −3.21, corresponding to a 64% reduction in microbial load. Further increasing the exposure time to 35 s led to an estimated value of −3.5 (19% reduction), indicating a slower inactivation rate at longer durations. Increased exposure to the pulsed electric field (PEF) raises the number of electric pulses, intensifying electroporation. This process forms temporary or permanent pores in the *E. coli* cell membrane, disrupting cellular balance and leading to bacterial inactivation. Studies have shown that longer treatment times and higher electric field strengths lead to greater microbial reduction (Krishnaveni 2023; Yeom et al. 2000). Treatment time has been found to be more influential than pulse frequency in reducing microbial populations (Mosqueda‐Melgar et al. 2007).

#### 3.2.2 Effect of Field Intensity and Nozzle Angle on Inactivation of *E. coli*

In addition to electric field intensity, another parameter influencing the reduction of microbial load in milk is the positioning angle of the nozzles in this pasteurization system. According to Figure 3b, increasing this angle from 0° to 45° corresponds to a 35% reduction in *Escherichia coli* in milk, lowering contamination from −2.38 to −3.21. Furthermore, increasing the nozzle angle up to 90° continues to decrease the microbial load in milk samples, reaching −2.73. However, this value indicates that beyond a certain point, further increases in angle lead to a slower rate of microorganism reduction. The nozzle angle in the combined ACP–PEF system affects how plasma jets are distributed and how effectively they interact with the milk. As indicated by the chart data, increasing the nozzle angle from 0° to 45° enables plasma jets to make more effective contact with the milk surface. This enhanced contact allows reactive oxygen and nitrogen species (RONS) such as ozone (O_3_) and hydroxyl radicals (·OH) to disperse more uniformly across the target liquid surface. This improved distribution promotes oxidation of the *E. coli* cell membrane, resulting in greater bacterial inactivation. As the nozzle positioning angle is further adjusted, when the angle reaches 90°, the plasma jets strike the milk in a more vertical direction. This reduces the effective contact area or leads to uneven dispersion of RONS within the liquid, which can in turn lower the efficiency of bacterial membrane oxidation. The findings of the present study regarding the importance of nozzle‐to‐sample distance are consistent with previous reports (Nishime et al. 2017).

In a study conducted by Baldanov et al. (2019), a cold plasma system using argon gas was employed to inactivate both Gram‐positive and Gram‐negative bacteria. The results demonstrated that the distance between the plasma jet and the microbial samples had a significant impact on microbial reduction and inactivation. Adjusting this distance from 0.5 to 3 cm led to considerable variations in the extent of microbial load reduction in the samples (Baldanov et al. 2019). Another study conducted by Lotfy (2020) further confirmed the importance of an optimal nozzle distance in ensuring effective reactive species distribution and enhancing plasma decontamination efficiency. In this work, a helium‐based plasma system was employed for the inactivation of *Escherichia coli* , with the nozzle‐to‐microbial sample distance optimally set and fixed at 7 mm, which yielded favorable results in microbial inactivation (Lotfy 2020).

On the other side of the chart, the reduction of microbial load under varying electric field intensities is shown. Comparing these two sections reveals that electric field intensity has a much greater and more pronounced effect on microbial load reduction. This claim is further supported by the coded Equation (4), which confirms that among all independent variables, nozzle angle has the least impact on *E. coli* reduction. Also, in the study by Gomez‐Gomez et al. (2021), electric field intensity was reported as one of the most important and influential parameters in microbial inactivation. An increase in this parameter led to a greater reduction in microbial load and contamination in the samples. According to the findings, an electric field intensity of 30 kV/cm enhanced *E. coli* inactivation by 0.2 and 0.5 log cycles compared to intensities of 25 and 20 kV/cm, respectively (Gomez‐Gomez et al. 2021).

#### 3.2.3 Effect of Exposure Time and Nozzle Angle on Inactivation of *E. coli*

Figure 3c illustrates the interaction effect of exposure time and nozzle positioning angle in the milk pasteurization system on the inactivation of *Escherichia coli* in milk. According to this figure, the decreasing trend on the right‐hand side, representing the effect of plasma treatment time on microbial load inactivation, shows a steeper slope, indicating a stronger influence. In contrast, the gentler slope on the left‐hand side, along with the coded equation (Equation 4), further confirms that the nozzle positioning angle has been less effective in reducing contamination in milk samples compared to the other independent variables in this experiment. In a study conducted by Jamali‐Hafshejani et al. (2025), it was demonstrated that the combined method of PEF‐ACP is an effective approach for the inactivation of *Escherichia coli* in sour cherry juice (Jamali‐Hafshejani et al. 2025).

According to the findings, among the parameters studied, the exposure time to the electric field had the most significant impact on microbial inactivation. Furthermore, the positioning of the plasma jets was found to be a critical factor in the process, necessitating the optimization of the nozzle arrangement. Ultimately, a nozzle angle of 3.44° was determined to ensure maximum contact between the plasma and the fluid surface.

#### 3.2.4 Effect of Nozzle Degree and Argon to Air Ratio on Inactivation of *E. coli*

Figure 3d illustrates the interactive effects of nozzle angle and argon‐to‐air ratio on *Escherichia coli* inactivation in milk using the combined cold PEF system. These variables were evaluated at three levels: nozzle angle (0°, 45°, and 90°) and argon‐to‐air ratio (0, 0.5, and 1). Based on the plot's slope and the coefficients of the coded equation (Equation 4), the argon‐to‐air ratio had a greater influence on microbial load reduction compared to nozzle angle. Analysis of Figure 3d revealed that increasing the argon‐to‐air ratio from 0 to 0.5 resulted in a logarithmic reduction from −2.82 to −3.21 in microbial load. However, at a ratio of 1 (pure argon), the logarithmic reduction was limited to approximately −2.0 (7% reduction), indicating lower efficiency and slower inactivation.

The argon‐to‐air ratio plays a critical role in plasma stability and the production of reactive oxygen and nitrogen species (RONS), such as ozone (O_3_) and hydroxyl radicals (·OH). Argon, as an inert gas, generates a stable electrical discharge and increases electron density, enhancing RONS production. These species oxidize the *E. coli* cell membrane, accelerating inactivation. However, at a ratio of 1 (pure argon), the lack of oxygen limits the production of oxygen‐based RONS, reducing inactivation efficiency. The optimal ratio balances plasma stability and RONS production, as sufficient oxygen supports oxidative reactions while argon maintains discharge stability (Bogusławska‐Wąs et al. 2023) (Misra et al. 2016).

### 3.3 Process Optimization

To achieve maximum *Escherichia coli* inactivation in milk, the combined ACP‐PEF process was optimized using RSM with a Box–Behnken design. The constraints for this optimization process are detailed in Table 2. The results identified the optimal conditions for microbial inactivation as an electric field strength of 10 kV/cm, an exposure time of 35 s, an argon‐to‐air ratio of 0.0 (pure air), and a nozzle angle of 53.87°. Under these conditions, the ACP‐PEF system achieved a logarithmic reduction of −4.65628 (99.9978% reduction) in *E. coli* microbial load.

**TABLE 2:** Optimization boundary conditions.

Name	Goal	Lower limit	Upper limit
Field intensity (kV/cm)	In range	5	10
Exposure time (s)	In range	5	35
Argon/air ratio	Minimize	0	1
Nozzle angle	In range	0	90
*E. coli* reduction (log (N/N_0_))	Minimize	−5.73	−1.22

### 3.4 Effect of the Combined PEF–ACP System on the Quality Properties of Milk at the Optimal Point

In this section, in order to assess the impact of the combined ACP‐PEF treatment on milk quality, key parameters including pH, fat content, protein content, lactose concentration, solids‐not‐fat (SnF), fat‐free portion (FFp), and color difference (ΔE) were evaluated. Comparisons were made between untreated milk, thermally pasteurized milk (conventional method), and ACP‐PEF‐treated milk under optimal conditions (electric field intensity of 10 kV/cm, exposure time of 35 s, argon‐to‐air ratio of 0.0, nozzle angle of 53.87°).

#### 3.4.1 Evaluation of pH, Fat, Protein, Lactose, SNF, and FFP of Milk in the ACP‐PEF System at the Optimal Point

Following treatment of milk with the ACP–PEF system under optimal conditions, measurements were conducted for pH, fat content, protein content, lactose, solids‐not‐fat (SNF), and free‐fat portion (FFP). These values were then compared with those of an untreated sample and a sample subjected to conventional thermal processing. The comparative data are summarized in Table 3.

**TABLE 3:** Effect of PEF‐ACP processing on quality properties of milk.

Property	Untreated milk	Suggested method	Conventional method
pH	6.75 ± 0.15a	6.72 ± 0.11a	6.60 ± 0.15a
Fat (%)	3.30 ± 0.15a	2.98 ± 0.17a	2.74 ± 0.15b
Protein (%)	3.48 ± 0.14a	3.21 ± 0.14a	3.05 ± 0.09b
Lactose (%)	5.01 ± 0.25a	4.680 ± 0.25a	4.40 ± 0.25a
Solids‐Not‐Fat (SNF)	9.20 ± 0.34a	8.81 ± 0.32a	8.1 ± 0.31b
Fat‐Free portion (FFP) (°C)	−0.537 ± 0.04a	−0.524 ± 0.07a	−0.528 ± 0.06a

Based on the obtained results, it can be concluded that applying the ACP–PEF system to milk under optimal conditions not only effectively inactivates *E. coli* but also results in fewer changes in the quality characteristics of milk compared to conventional thermal treatment. Specifically, ACP–PEF treatment led to reductions of 0.44% in pH, 10% in fat, 7% in protein and lactose, 4% in SNF, and 2% in FFP compared to the untreated sample. In contrast, thermally treated milk (treated with conventional methods) showed reductions of 2% in pH, 20% in fat, 12% in protein, 12% in lactose, 12% in SNF, and 1.67% in FFP, respectively, compared to the untreated sample.

Overall, the combined ACP–PEF system, under optimal conditions, not only effectively inactivates *Escherichia coli* but also better preserves the quality parameters of milk compared to conventional thermal pasteurization. The nonthermal nature of this system, utilizing PEF‐induced electroporation and reactive oxygen and nitrogen species (RONS) generated by cold plasma, minimizes the chemical and physical alterations in milk.

Milk pH is a key indicator of its quality and stability. Since the designed pasteurization system operates through a non‐thermal approach, it has successfully maintained the pH of milk samples. Moreover, ACP–PEF has minimal impact on fat globules, as PEF and RONS primarily target microbial cell membranes rather than lipids. In contrast, thermal pasteurization induces lipid oxidation and alters flavor.

The absence of heating in ACP–PEF also prevents protein denaturation, thereby maintaining protein solubility and digestibility. Thermal pasteurization, however, promotes protein aggregation and reduces functionality. This advantage of ACP–PEF contributes to the superior preservation of milk's nutritional value. Another key benefit of this non‐thermal method is the prevention of Maillard reactions and heat‐induced lactose degradation, which helps retain the sweetness and energy content of milk. In contrast, thermal processes degrade lactose, negatively affecting flavor and stability.

The minimal effect of ACP–PEF on SNF further indicates the preservation of soluble components and minerals in milk, unlike thermal pasteurization, which causes losses of these constituents and reduces nutritional quality.

In a study conducted by Taki et al. (2025) cow's milk was pasteurized using a plasma‐assisted non‐thermal method. The findings of that study were consistent with those of the present research, demonstrating that plasma treatment and other non‐thermal approaches offer advantages over conventional thermal methods by providing superior preservation of milk's quality and nutritional properties while ensuring complete elimination of contaminants and pathogens (Taki et al. 2025) (Segat et al. 2015) (Jamali‐Hafshejani et al. 2025).

#### 3.4.2 Color Evaluation

In this study, the ΔE value, which indicates the overall color difference between the control sample and the treated samples using both conventional thermal methods and the non‐thermal combined pulsed electric field‐cold plasma method, was calculated. The color evaluation of the milk samples showed no significant difference in color between the control sample and the sample treated with the PEF‐ACP pasteurization system at the optimal experimental point. The results of this evaluation are presented in Table 4. Analytical measurements confirmed that the color parameters L*, a*, and b*, as well as the total color difference (ΔE), exhibit very minor differences between the control samples and those treated with the combined system. This indicates that the treatment with the designed combined pasteurization system in this experiment has a very minimal impact on milk quality, which will not affect the marketability of the product and preserve the final product quality.

**TABLE 4:** Colorimetric parameters of treated and untreated milk.

Samples	Hunter color value			
	l*	a*	b*	ΔE
Control sample	88.2 ± 0.5a	−0.90 ± 0.1a	4.40 ± 0.15a	—
Treated sample (with combined system)	87.4 ± 0.5a	0.87 ± 0.14a	4.25 ± 0.16ab	0.81 ± 0.10a
Treated sample (with conventional method)	86.3 ± 0.5b	0.81 ± 0.41a	4.19 ± 0.11b	1.91 ± 0.18b

*Note:* *indicates difference letters which shows significant effect.

According to this Table, the color difference between the control sample and the sample treated with the designed combined pasteurization system is 0.81, which falls within the desirable range and is not visible to the human eye. In contrast, examining the reported value for the color difference between the sample treated with conventional thermal methods and the control sample (1.91) reveals a slightly higher but still acceptable color difference in this case.

Previous research findings, including the study by Gurol et al. (2012), have also shown that cold plasma treatment has no effect on the color of milk samples (Gurol et al. 2012). Additionally, the research by Wu et al. (2021) demonstrated that applying plasma for 120 s to milk samples results in an acceptable color difference within the desirable range (Wu et al. 2021). However, excessive plasma application may cause changes in sample color; a study by Gurol et al. (2012) also proved that 20 min of plasma treatment has a significant impact on the color of milk samples (Gurol et al. 2012). In another study conducted by Jamali‐Hafshejani et al. (2025), it was shown that the pulsed electric field‐cold plasma system in sour cherry juice samples reported a 2.3% reduction in lightness (L*), 1.7% reduction in redness (a*), and 1.5% reduction in yellowness (b*). In contrast, using conventional thermal pasteurization methods showed reductions of 9.6%, 3.5%, and 4.5% in lightness, redness, and yellowness, respectively (Jamali‐Hafshejani et al. 2025). It can be concluded that treatment with the PEF‐ACP system, compared to conventional thermal methods, has no adverse effect on the color of the samples, and this pasteurization system, in addition to its ability to reduce microbial contamination, can effectively maintain the quality of the final product.

## 4 Conclusion

This study successfully developed and optimized an ACP‐PEF system for non‐thermal milk pasteurization, achieving a −4.656‐log reduction in *E. coli* under optimal conditions (10 kV/cm, 35 s, argon‐to‐air ratio 0, nozzle angle 53.87°). The RSM model demonstrated exceptional accuracy (*R* ^2^ = 0.9997), confirming the significant influence of electric field intensity and exposure time on microorganism inactivation. The non‐thermal approach minimized quality changes, with pH, fat, protein, lactose, SNF, and FFP reductions of 0.44%–10%, far less than thermal pasteurization (2%–20%). Color preservation (ΔE = 0.81) further highlights the system's superiority in maintaining sensory attributes. The specific energy consumption for the ACP‐PEF system at optimal conditions was 120 kJ/L, which is comparable to conventional HTST pasteurization, which typically consumes 100–150 kJ/L. The synergy between PEF's electroporation and ACP's RONS production enables efficient microbial inactivation without heat‐related degradation, addressing limitations of conventional methods. This innovation holds promise for the dairy industry, offering a sustainable, scalable solution for producing microbiologically safe milk with retained nutritional value. Overall, ACP‐PEF represents a breakthrough in non‐thermal processing, enhancing food safety and quality in dairy products.

## Author Contributions

K.T.: Investigation, methodology, writing – original draft, review‐editing. B.H.S.: Conceptualization, project administration, supervision, writing – review and editing. S.G.: Writing – reviewing and editing.

## Conflicts of Interest

The authors declare no conflicts of interest.

## Data Availability

The data that support the findings of this study are available from the corresponding author upon reasonable request.
